# Lower gut abundance of *Eubacterium rectale* is linked to COVID-19 mortality

**DOI:** 10.3389/fcimb.2023.1249069

**Published:** 2023-09-06

**Authors:** Yingzhi Liu, Matthew T. V. Chan, Francis K. L. Chan, William K. K. Wu, Siew C. Ng, Lin Zhang

**Affiliations:** ^1^ Microbiota I-Center (MagIC), Hong Kong, Hong Kong SAR, China; ^2^ Department of Anaesthesia and Intensive Care and Peter Hung Pain Research Institute, Faculty of Medicine, The Chinese University of Hong Kong, Hong Kong, Hong Kong SAR, China; ^3^ Centre for Gut Microbiota Research, The Chinese University of Hong Kong, Hong Kong, Hong Kong SAR, China; ^4^ State Key Laboratory of Digestive Disease, Li Ka Shing Institute of Health Sciences, Institute of Digestive Disease, The Chinese University of Hong Kong, Hong Kong, Hong Kong SAR, China; ^5^ Department of Medicine and Therapeutics, Faculty of Medicine, The Chinese University of Hong Kong, Hong Kong, Hong Kong SAR, China

**Keywords:** gut microbiome, *Eubacterium rectale*, COVID-19, mortality, population based

## Abstract

**Introduction:**

Emerging preclinical and clinical studies suggest that altered gut microbiome composition and functions are associated with coronavirus 2019 (COVID- 19) severity and its long-term complications. We hypothesize that COVID-19 outcome is associated with gut microbiome status in population-based settings.

**Methods:**

Gut metagenomic data of the adult population consisting of 2871 subjects from 16 countries were obtained from ExperimentHub through R, while the dynamic death data of COVID-19 patients between January 22, 2020 and December 8, 2020 in each country was acquired from Johns Hopkins Coronavirus Resource Center. An adjusted stable mortality rate (SMR) was used to represent these countries’ mortality and correlated with the mean relative abundance (mRA) of healthy adult gut microbiome species.

**Results:**

After excluding bacterial species with low prevalence (prevalence <0.2 in the included countries), the β-diversity was significantly higher in the countries with high SMR when compared with those with median or low SMR (p <0.001). We then identified the mRA of two butyrate producers, *Eubacterium rectale* and *Roseburia intestinalis*, that were negatively correlated with SMR during the study period. And the reduction of these species was associated with severer COVID-19 manifestation.

**Conclusion:**

Population-based microbiome signatures with the stable mortality rate of COVID-19 in different countries suggest that altered gut microbiome composition and functions are associated with mortality of COVID-19.

## Main text

A previous cohort study showed that the gut microbial diversity was altered in COVID-19-infected subjects (Zhang et al., 2023). Likewise, a previous study suggested that the microbiome change in COVID-19 patients was driven by the enrichment of *Ruminococcus gnavus*, *Ruminococcus torques*, and *Bacteroides dorei*, and the depletion of beneficial bacterial species, including *Bifidobacterium adolescentis*, *Faecalibacterium prausnitzii*, and *Eubacterium rectale* (*E. rectale*) (Yeoh et al., 2021). However, whether COVID-19 outcome is associated with pre-existing gut microbiome status in population-based settings is unknown. Herein, gut metagenomic data of the adult population consisting of 2,871 subjects from 16 countries were obtained from ExperimentHub (**STable 1**, **SFigure 1A**), and the dynamic incidence and mortality of COVID-19 between January 22, 2020 and December 8, 2020 in each country were obtained from Johns Hopkins Coronavirus Resource Center (**SFigure 1A**) (Pasolli et al., 2017; Dong et al., 2020). An adjusted stable mortality rate (SMR, **SFigure 1A**) was used to indicate the mortality rate in these countries (**SFigure 1B**), and we correlated mortality data with the mean relative abundance (mRA) of healthy adult gut bacterial species. We chose the longest duration of the stable period before the introduction of the vaccination programme for all countries to calculate SMR, through which we uncovered the relationship between microbiota profiles and the SMR of COVID-19 (**SFigure 1A**).

Overall, although the α-diversity of the gut microbiota did not show any difference, the inverse Simpson (1-Simpson) had a marginal *p*-value (*p* = 0.054) (**SFigure 1C**). β-diversity was significantly higher in the countries with high SMR when compared with those with median or low SMR (*p <*0.001) (**SFigure 1D**). Importantly, after excluding bacterial species with low prevalence rates (<0.2 in the above countries), half of the top 20 bacteria (**STable 2**) that showed negative correlations with SMR were butyrate producers (highlighted in green).

**Figure 1:**
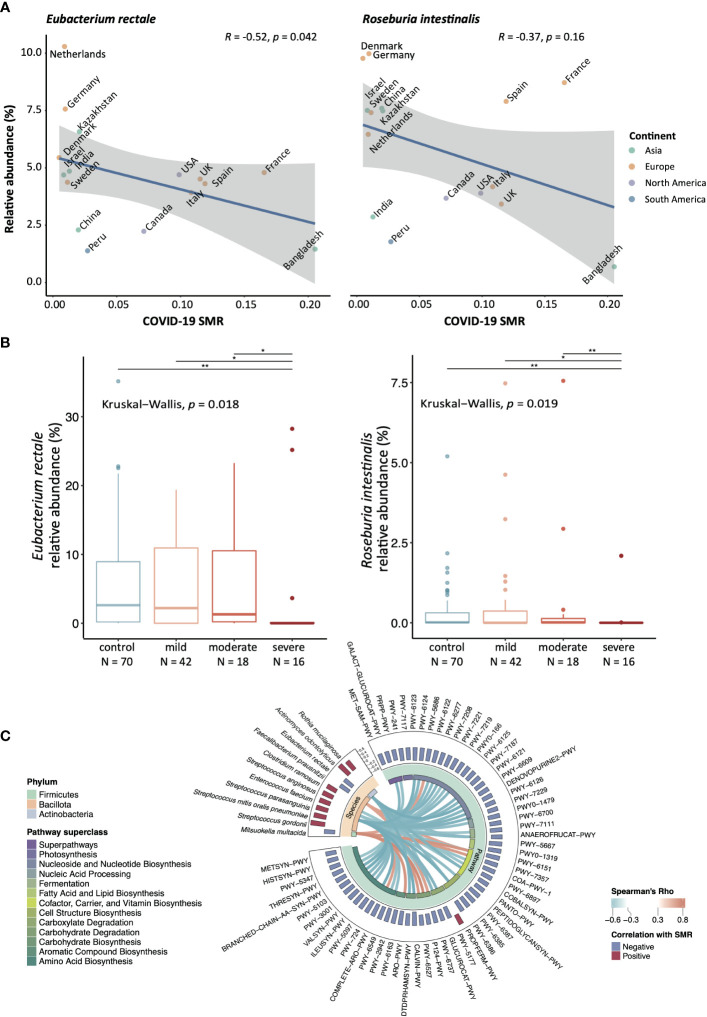
The bacterial species that correlate with SMR amongst different countries Significance level: *:*p*-value <0.05, **p-value <0.01. **(A)** Correlation between mRA and SMR in *E. rectale* and *R. intestinalis*, two-sided Spearman correlation test has been performed. **(B)** The validation cohort from Hong Kong COVID-19 cohort (Severe N = 16, moderate N = 18, mild = 42, control = 70). The relative abundance of *E. rectale* and *R. intestinalis* from the fecal metagenomic data were compared with the Kruskal−Wallis test and *post-hoc* tested with Wilcox rank-sum test amongst each disease/control group. The reduction of relative abundance of *E. rectale* in human fecal samples was reported in COVID-19 patients from the same cohort (Yeoh et al., 2021) **(C)** The correlations between SMR-correlated functional pathways and bacteria species were conducted using Hierarchical All-against-All association testing (HAllA), and only species/pathways with at least one significant correlation were shown.

The Omicron variant has caused an unprecedented pandemic with distinct phenotypes, and COVID-related mortality has dropped significantly with an explosion in infection rate (**STable 3**) (Hoffmann et al., 2022; Nyberg et al., 2022). Therefore, we applied the same analytic strategy to the Omicron pandemic to determine the replicability of our findings. Four overlapped species among the top-20s were identified, namely *E. rectale*, *Roseburia intestinalis* (*R. intestinalis*), *Bifidobacterium angulatum*, and *Parabacteroides* unclassified (**Figure 1A**, **STable 2**). It should be noted that a well-known beneficial butyrate producer, *E. rectale*, was the only species that was correlated significantly with the mortality outcome of all SARS-CoV-2 variants, i.e., the Alpha, Beta, Gamma variants, and the Omicron variant (**STable 2**) (Louis and Flint, 2009; Zhang et al., 2022). To validate the findings generated from the public dataset, we determined the relative abundance of the four species in the published Hong Kong COVID-19 cohorts that was conducted before the introduction of Hong Kong’s vaccination programme (Zuo et al., 2020; Yeoh et al., 2021; Zhang et al., 2022). From **Figure 1B** we found that the relative abundance of *E. rectale* and *R. intestinalis* was much lower in patients with severe COVID-19 compared to the control subjects or COVID-19 patients with mild or moderate symptoms.

Moreover, we identified 181 functional pathways and eleven bacteria species that were significantly correlated with SMR (**STables 2, 4**). To pinpoint the associated features between these bacterial species or functional pathways related to SMR, we performed all-against-all association testing to identify the adjusted correlations. **Figure 1C** presents the association between the differential species and pathways. Interestingly, the species negatively correlated with SMR were significantly positively correlated with the depleted pathways under the superclasses of 1) carbohydrate degradation (PWY-6737, P124-PWY, and PWY-6527); 2) cofactor, carrier, and vitamin biosynthesis (PWY-6151, PWY-7357) (**Figure 1C**), indicating that these functional deficiencies of the gut microbiota at the baseline might be linked to higher COVID-19 mortality.

## Discussion

In this study, for the first time, we identified a pre-existence mRA of *E. rectale* and *R. intestinalis* in a healthy population before the COVID-19 pandemic are associated with lower COVID-19 regional mortality in a population-based gut microbiota study. The depletion of both identified species, *E. rectale* and *R. intestinalis* have been reported not only in COVID-19 (Zuo et al., 2020; Cao et al., 2021; Yeoh et al., 2021; Zhang et al., 2022) but also in ulcerative colitis patients (Pittayanon et al., 2020; Shen et al., 2022) and is possibly linked to the reduction of host inflammatory response. The diminished abundance of *E. rectale* comes with the negative correlation to C-X-C motif ligand 10 (CXCL10) and tumor necrosis factor-alpha (TNF-α), two inflammation markers that indicate the origination of immune response at the early stage of COVID-19 (Yeoh et al., 2021). *R. intestinalis* inhibits the development of Crohn’s disease (CD) by increasing the differentiation of anti-inflammatory Tregs, which may provide the basis of new therapeutic strategies for CD (Shen et al., 2022). Such a decrease of butyrate-producing taxa, such as *Blautia* and *Eubacterium (rectale)*, in influenza A virus infection was proven in the previous 16s studies (Fuentes et al., 2021; Bhar et al., 2022). Strikingly, in the pathway analysis, results displayed a strong association between the depleted species and reduced pathways, implying that the protective role of the gut microbiome in the population could be caused by their biosynthesis functions. Of them, the carbohydrate degradation pathways could be mainly contributed by butyrate-producing species (Flint et al., 2012) with the fermentation of carbohydrates.

There were several limitations of this study. First, the mechanism between gut microorganisms and immune functions was not further explored in the study, and it would be beneficial to understand how microbiota derive metabolites or immune activation against infection for future applications in medication or prevention. In addition, due to the limitation of the online metagenomic database, the study did not consider antibiotic usage at an individual or regional level, which could mask the metagenomic profiling and mortality rate in a country. The alteration of the bacterial community structure after antibiotic treatment has been reported (Hill et al., 2010). On the other hand, long-term antibiotic exposure may be a risk factor for all-cause mortality (Heianza et al., 2020; Verdecchia et al., 2020). Another disadvantage is that the study applied an imbalanced sample size from different countries to represent the populational species’ relative abundance. Unlike epidemiology-based cohorts, due to the heterogeneity of each country and individual, metagenomic-based cohorts, restricted by their expense and feasibility, could only serve as a nation miniature. As a compromise, we conducted the validation cohort in the Hong Kong population to confirm our findings. Yet, a more extensive study with larger sample sizes is required to validate the predictive capability of these two butyrate producers. Third, this study considered COVID-19 mortality as the only outcome, which may neglect that the low abundance of *E. rectale* may also imply the association with other disease conditions (Vermeiren et al., 2012; Zhu et al., 2018; Su et al., 2022). Therefore, although the metadata from the public datasets was limited, all the included subjects were labelled as a healthy condition to eliminate the potential bias. To our best knowledge, there are lack of comparative metagenomic-based gut microbiome-related studies related to other emerging infectious diseases at the time of our study endpoint. However, the reduction of beneficial microbial products, especially butyrate, in influenza A virus infection and sepsis has been described (Adelman et al., 2020; Sencio et al., 2020).

Presenting the negative association between the populational gut abundance of two butyrate producers and the COVID-19 regional mortality, our study offered hope that microbiota modulation could be one of the keys to reducing COVID-19-related mortality. In particular, to develop butyrate-producing probiotics with high-fibber diet may assist the enrichment of such beneficial species (Kasahara et al., 2018). Nonetheless, future assessment of these potential next-generation probiotics in animal models of COVID-19 and clinical trials in humans is warranted.

## Data availability statement

The original contributions presented in the study are included in the article/**Supplementary Material**. Further inquiries can be directed to the corresponding authors.

## Ethics statement

Ethical approval was not required for the study involving humans in accordance with the local legislation and institutional requirements. Written informed consent to participate in this study was not required from the participants or the participants’ legal guardians/next of kin in accordance with the national legislation and the institutional requirements.

## Author contributions

LZ, WW, MC, FC and SN conceived and designed the study. WW, LZ and YL developed the detailed methodology. LZ and YL were responsible for data collection and data analysis, interpreted the results, and drafted the manuscript. WW, SN, MC and FC reviewed the manuscript with critical suggestions. All authors critically reviewed and approved the final version. All authors had full access to all the data in the study and accepted the responsibility to submit it for publication. LZ and SN had final responsibility for the decision to submit for publication. All authors contributed to the article and approved the submitted version.

## References

[B1] Adelman M. W. Woodworth M. H. Langelier C. Busch L. M. Kempker J. A. Kraft C. S.. (2020). The gut microbiome’s role in the development, maintenance, and outcomes of sepsis. Crit. Care 24, 278. doi: 10.1186/s13054-020-02989-1 32487252 PMC7266132

[B2] Bhar A. Gierse L. C. Meene A. Wang H. Karte C. Schwaiger T.. (2022). Application of a maximal-clique based community detection algorithm to gut microbiome data reveals driver microbes during influenza A virus infection. Front. Microbiol. 13. doi: 10.3389/fmicb.2022.979320 PMC9630851 36338082

[B3] Cao J. Wang C. Zhang Y. Lei G. Xu K. Zhao N.. (2021). Integrated gut virome and bacteriome dynamics in COVID-19 patients. Gut Microbes 13, 1–21. doi: 10.1080/19490976.2021.1887722 PMC7946006 33678150

[B4] Dong E. Du H. Gardner L. (2020). An interactive web-based dashboard to track COVID-19 in real time. Lancet Infect. Dis. 20, 533–534. doi: 10.1016/S1473-3099(20)30120-1 32087114 PMC7159018

[B5] Flint H. J. Scott K. P. Duncan S. H. Louis P. Forano E. (2012). Microbial degradation of complex carbohydrates in the gut. Gut Microbes 3, 289–306. doi: 10.4161/gmic.19897 22572875 PMC3463488

[B6] Fuentes S. den Hartog G. Nanlohy N. M. Wijnands L. Ferreira J. A. Nicolaie M. A.. (2021). Associations of faecal microbiota with influenza-like illness in participants aged 60 years or older: an observational study. Lancet Healthy Longev. 2, e13–e23. doi: 10.1016/S2666-7568(20)30034-9 36098111

[B7] Heianza Y. Ma W. Li X. Cao Y. Chan A. T. Rimm E. B.. (2020). Duration and life-stage of antibiotic use and risks of all-cause and cause-specific mortality: prospective cohort study. Circ. Res. 126, 364–373. doi: 10.1161/CIRCRESAHA.119.315279 31842690 PMC7046316

[B8] Hill D. A. Hoffmann C. Abt M. C. Du Y. Kobuley D. Kirn T. J.. (2010). Metagenomic analyses reveal antibiotic-induced temporal and spatial changes in intestinal microbiota with associated alterations in immune cell homeostasis. Mucosal Immunol. 3, 148–158. doi: 10.1038/mi.2009.132 19940845 PMC2824244

[B9] Hoffmann M. Kruger N. Schulz S. Cossmann A. Rocha C. Kempf A.. (2022). The Omicron variant is highly resistant against antibody-mediated neutralization: Implications for control of the COVID-19 pandemic. Cell 185, 447–456 e411. doi: 10.1016/j.cell.2021.12.032 35026151 PMC8702401

[B10] Kasahara K. Krautkramer K. A. Org E. Romano K. A. Kerby R. L. Vivas E. I.. (2018). Interactions between Roseburia intestinalis and diet modulate atherogenesis in a murine model. Nat. Microbiol. 3, 1461–1471. doi: 10.1038/s41564-018-0272-x 30397344 PMC6280189

[B11] Louis P. Flint H. J. (2009). Diversity, metabolism and microbial ecology of butyrate-producing bacteria from the human large intestine. FEMS Microbiol. Lett. 294, 1–8. doi: 10.1111/j.1574-6968.2009.01514.x 19222573

[B12] Nyberg T. Ferguson N. M. Nash S. G. Webster H. H. Flaxman S. Andrews N.. (2022). Comparative analysis of the risks of hospitalisation and death associated with SARS-CoV-2 omicron (B.1.1.529) and delta (B.1.617.2) variants in England: a cohort study. Lancet 399, 1303–1312. doi: 10.1016/S0140-6736(22)00462-7 35305296 PMC8926413

[B13] Pasolli E. Schiffer L. Manghi P. Renson A. Obenchain V. Truong D. T.. (2017). Accessible, curated metagenomic data through ExperimentHub. Nat. Methods 14, 1023–1024. doi: 10.1038/nmeth.4468 29088129 PMC5862039

[B14] Pittayanon R. Lau J. T. Leontiadis G. I. Tse F. Yuan Y. Surette M.. (2020). Differences in gut microbiota in patients with vs without inflammatory bowel diseases: a systematic review. Gastroenterology 158, 930–946 e931. doi: 10.1053/j.gastro.2019.11.294 31812509

[B15] Sencio V. Barthelemy A. Tavares L. P. Machado M. G. Soulard D. Cuinat C.. (2020). Gut Dysbiosis during Influenza Contributes to Pulmonary Pneumococcal Superinfection through Altered Short-Chain Fatty Acid Production. Cell Rep. 30, 2934–2947 e2936. doi: 10.1016/j.celrep.2020.02.013 32130898

[B16] Shen Z. Luo W. Tan B. Nie K. Deng M. Wu S.. (2022). Roseburia intestinalis stimulates TLR5-dependent intestinal immunity against Crohn’s disease. EBioMedicine 85, 104285. doi: 10.1016/j.ebiom.2022.104285 36182776 PMC9526137

[B17] Su Q. Liu Q. Lau R. I. Zhang J. Xu Z. Yeoh Y. K.. (2022). Faecal microbiome-based machine learning for multi-class disease diagnosis. Nat. Commun. 13, 6818. doi: 10.1038/s41467-022-34405-3 36357393 PMC9649010

[B18] Verdecchia P. Angeli F. Cavallini C. Reboldi G. (2020). Use of antibiotics and mortality in women: does duration of exposure matter? Circ. Res. 126, 374–376. doi: 10.1161/CIRCRESAHA.119.316406 31999533

[B19] Vermeiren J. Van den Abbeele P. Laukens D. Vigsnaes L. K. De Vos M. Boon N.. (2012). Decreased colonization of fecal Clostridium coccoides/Eubacterium rectale species from ulcerative colitis patients in an in *vitro* dynamic gut model with mucin environment. FEMS Microbiol. Ecol. 79, 685–696. doi: 10.1111/j.1574-6941.2011.01252.x 22092917

[B20] Yeoh Y. K. Zuo T. Lui G. C. Zhang F. Liu Q. Li A. Y.. (2021). Gut microbiota composition reflects disease severity and dysfunctional immune responses in patients with COVID-19. Gut 70, 698–706. doi: 10.1136/gutjnl-2020-323020 33431578 PMC7804842

[B21] Zhang F. Wan Y. Zuo T. Yeoh Y. K. Liu Q. Zhang L.. (2022). Prolonged impairment of short-chain fatty acid and L-isoleucine biosynthesis in gut microbiome in patients with COVID-19. Gastroenterology 162, 548–561 e544. doi: 10.1053/j.gastro.2021.10.013 34687739 PMC8529231

[B22] Zhang F. Lau R. I. Liu Q. Su Q. Chan F. K. L. Ng S. C. (2023). Gut microbiota in COVID-19: key microbial changes, potential mechanisms and clinical applications. Nat. Rev. Gastroenterol. Hepatol. 20, 323–337. doi: 10.1038/s41575-022-00698-4 36271144 PMC9589856

[B23] Zhu Q. Gao R. Zhang Y. Pan D. Zhu Y. Zhang X.. (2018). Dysbiosis signatures of gut microbiota in coronary artery disease. Physiol. Genomics 50, 893–903. doi: 10.1152/physiolgenomics.00070.2018 30192713

[B24] Zuo T. Zhang F. Lui G. C. Y. Yeoh Y. K. Li A. Y. L. Zhan H.. (2020). Alterations in gut microbiota of patients with COVID-19 during time of hospitalization. Gastroenterology 159, 944–955 e948. doi: 10.1053/j.gastro.2020.05.048 32442562 PMC7237927

