# Supplementary material for: Lower gut abundance of Eubacterium rectale is linked to COVID-19 mortality
Source: Front Cell Infect Microbiol. 2023 Sep 6;13:1249069. doi: 10.3389/fcimb.2023.1249069 (PMC10512258; doi:10.3389/fcimb.2023.1249069)
Supplement: Supplementary file 1 [file DataSheet_1.pdf]

Supplementary Figure 1

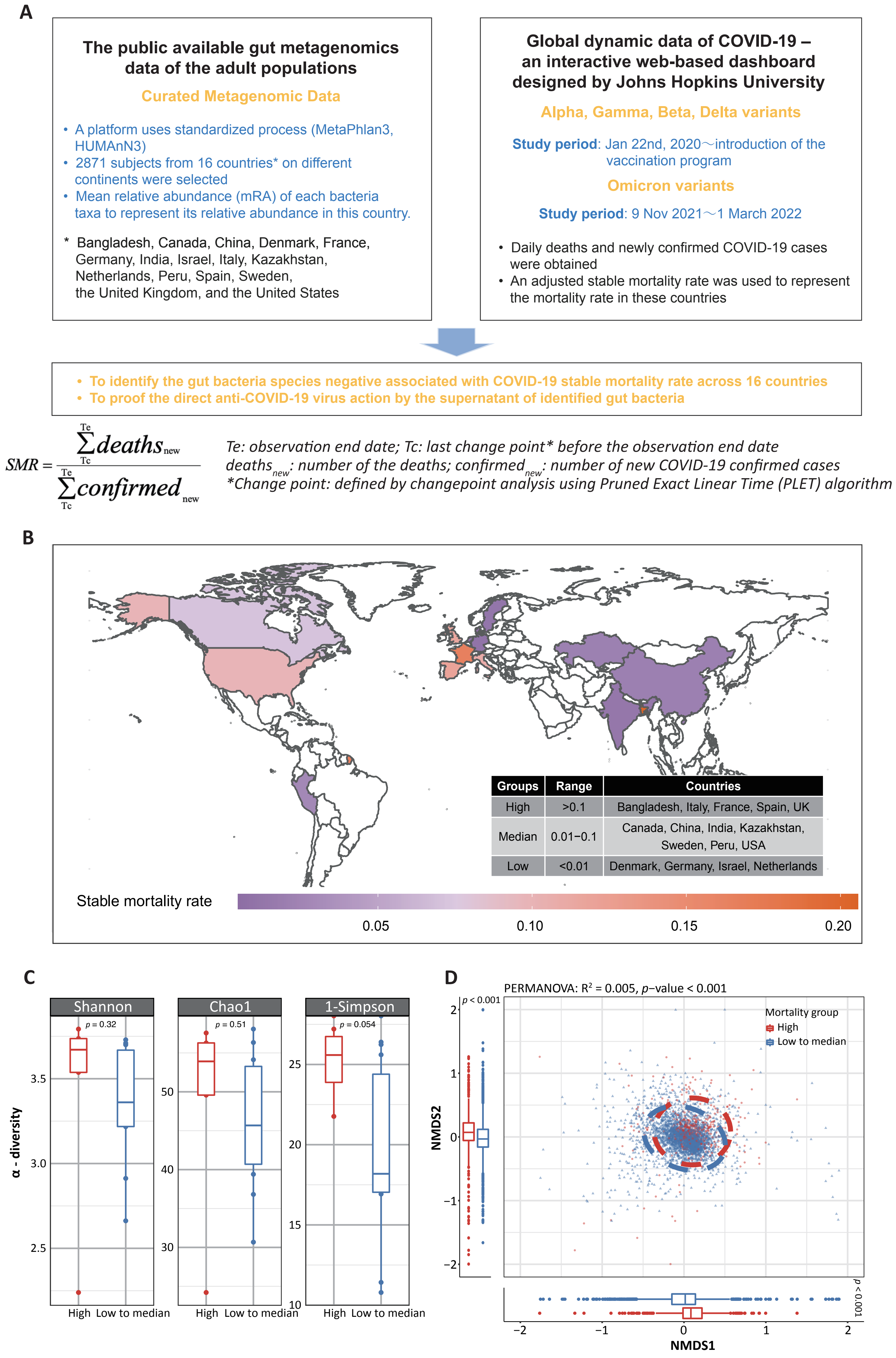

Supplementary Figure 1 An overview of the included cohorts. **(A)** Detailed cohort information, cohort period definition, aims, and the formula of SMR calculation. **(B)** SMR group definition and its distribution among 16 selected countries. **(C)** Individual metagenomic data were used to compare the α diversity in counties in different SMR groups with Wilcox rank-sum test. **(D)** β diversity was presented with non-metric Multidimensional Scaling (NMDS), while the p-value was given by permutational multivariate analysis of variance (PERMANOVA).

Supplementary methods

Data acquisition and taxonomic data preprocessing

1. Metagenomic data of healthy adults

We obtained the processed metagenomic data using the “curatedMetagenomicData” package from R, which uses a standardized protocol (MetaPhlan3 and HUMAnN3) to analyze published metagenomic data and creates consolidated datasets. In this analysis, data from 16 countries (Bangladesh, Canada, China, Denmark, France, Germany, India, Israel, Italy, Kazakhstan, Netherlands, Peru, Spain, Sweden, the United Kingdom, and the United States) were downloaded, and the earliest OTU record during the study period was kept representing each subject for analysis. Due to lacking sharing overlap co-variates in the metadata from the all the studies in the database, only age, gender, specimen type, and disease status were included in this cohort for further sample selection. Stool samples that were labeled with healthy in disease status and age category identified as adult were included. The final sample size and relevant cohorts were recorded in the **STable 1**. A total of 2871 unique records from healthy adult stool samples were selected and combined into a new dataset. On the basis of the previously used method, we calculated the mean relative abundance (mRA) of each bacterial taxa from each country to serve as its relative abundance in this country.

2. Global data of COVID-19

The dynamic data of diagnosis and death data of COVID-19 patients in each country between January 22, 2020 and the introduction of the vaccination program of each country was acquired from COVID-19 Dashboard, an interactive web-based dashboard that was established by the Center for Systems Science and Engineering of Johns Hopkins University (https://github.com/CSSEGISandData/COVID-19/tree/web-data). The daily deaths and newly confirmed COVID-19 cases were reported rely on local health departments and local media reports.

Stable mortality rate (SMR)

We applied R package “changepoint.np”, a package that was designed for changepoint analysis using Pruned Exact Linear Time (PLET) algorithm, to detect the change point (T_c) of DR_{14}.[1] Non-parametric approach with two integers as minimum segment length and quantiles equal to 2*log2 (number of valid days) was set up for the T_c detection. Next, among the several T_cs that detected during the COVID pandemic period and before the introduction day of the vaccination program of each country, we chose the longest duration (T_c to T_{c+1}) as stable period and set the T_{c+1} as T_e. Finally, we calculated the SMR based on the above parameters and the below formulas (**SFigure 1A**).

$$SMR = \frac{\sum_{Tc}^{Te} deaths_{new}}{\sum_{Tc}^{Te} confirmed_{new}}$$

Other statistical analysis

All statistical analysis was conducted in R version 4.2.1 (RProject for Statistical Computing). A nominal p-value less than 0.05 was considered statistically significant. The correlation between two continuous variables was calculated by Spearman (two-sided) test with the "corr" package. The bacterial species/functional pathway with a 0 mRA in more than 20% of the included countries has been removed from the correlation tests. Non-metric multidimensional scaling (NMDS) and the analysis of similarities (ANOSIM) with the Bray-Curtis dissimilarity approach were used to determine the group differences of the β-diversity amongst different SMR groups. α-diversity indices, including richness, Shannon and Simpson index, were calculated based on the species profile for each sample. Similar to mRA, the α-diversity of each country also presented with individual means. The correlations between bacterial species and functional pathways were conducted using Hierarchical All-against-All association testing (HAllA), a computational method to find multi-resolution associations in high-heterogeneous datasets.[2] Other packages for the analyses and data visualization were: phyloseq, vegan, ComplexHeatmap, circlize, tidyverse, hrbrthemes, dplyr, glm, ppcor, pROC, ggplot2, ggpubr and Hmisc.

Validation cohorts

We evaluate the predictive value of selected bacterial species to the clinical outcomes using Hong Kong local cohort. Baseline individual relative abundance were compared within different severity groups among COVID patients, and the detailed subject recruitment and sample collection methods have been descripted previously.[3-5]

1. Killick, R. and I.A. Eckley, *changepoint: An R Package for Changepoint Analysis.* Journal of Statistical Software, 2014. **58**(3): p. 1 - 19.
2. Ghazi, A.R., et al., *High-sensitivity pattern discovery in large, paired multiomic datasets.* Bioinformatics, 2022. **38**(Suppl 1): p. i378-i385.
3. Yeoh, Y.K., et al., *Gut microbiota composition reflects disease severity and dysfunctional immune responses in patients with COVID-19.* Gut, 2021. **70**(4): p. 698-706.
4. Zhang, F., et al., *Prolonged Impairment of Short-Chain Fatty Acid and L-Isoleucine Biosynthesis in Gut Microbiome in Patients With COVID-19.* Gastroenterology, 2022. **162**(2): p. 548-561 e4.
5. Zuo, T., et al., *Alterations in Gut Microbiota of Patients With COVID-19 During Time of Hospitalization.* Gastroenterology, 2020. **159**(3): p. 944-955 e8.
